# Interannual temperature variability is a principal driver of low-frequency fluctuations in marine fish populations

**DOI:** 10.1038/s42003-021-02960-y

**Published:** 2022-01-11

**Authors:** Peter van der Sleen, Pieter A. Zuidema, John Morrongiello, Jia Lin J. Ong, Ryan R. Rykaczewski, William J. Sydeman, Emanuele Di Lorenzo, Bryan A. Black

**Affiliations:** 1 Wildlife Ecology and Conservation Group, Wageningen University and Research Centre, Droevendaalsesteeg 3a, 6708 PB Wageningen, The Netherlands; 2 Forest Ecology and Forest Management Group, Wageningen University and Research Centre, Droevendaalsesteeg 3a, 6708 PB Wageningen, The Netherlands; 3 School of BioSciences, The University of Melbourne, Melbourne, VIC 3010 Australia; 4 Asian School of the Environment, Nanyang Technological University, Singapore, 639798 50 Nanyang Avenue, Block N2-01C-48, Singapore; 5 Ecosystem Sciences Division, NOAA Pacific Islands Fisheries Science Center, 1845 Wasp Blvd, Bldg. 176, Honolulu, HI 96818-5007 USA; 6 Farallon Institute, 101 H Street, Suite Q, Petaluma, CA 94952 USA; 7 School of Earth and Atmospheric Sciences, Georgia Institute of Technology, 311 Ferst Drive NE, Atlanta, GA 30306 USA; 8 Laboratory of Tree-Ring Research, University of Arizona, 1215 E Lowell St, Tucson, AZ 85721 USA

**Keywords:** Fisheries, Population dynamics, Stochastic modelling, Climate-change ecology

## Abstract

Marine fish populations commonly exhibit low-frequency fluctuations in biomass that can cause catch volatility and thus endanger the food and economic security of dependent coastal societies. Such variability has been linked to fishing intensity, demographic processes and environmental variability, but our understanding of the underlying drivers remains poor for most fish stocks. Our study departs from previous findings showing that sea surface temperature (SST) is a significant driver of fish somatic growth variability and that life-history characteristics mediate population-level responses to environmental variability. We use autoregressive models to simulate how fish populations integrate SST variability over multiple years depending on fish life span and trophic position. We find that simulated SST-driven population dynamics can explain a significant portion of observed low-frequency variability in independent observations of fisheries landings around the globe. Predictive skill, however, decreases with increasing fishing pressure, likely due to demographic truncation. Using our modelling approach, we also show that increases in the mean and variance of SST could amplify biomass volatility and lessen its predictability in the future. Overall, biological integration of high-frequency SST variability represents a null hypothesis with which to explore the drivers of low-frequency population change across upper-trophic marine species.

## Abstract (web summary)

van der Sleen et al. introduce the use of an autoregressive null model to explain low-frequency variability in populations of marine fishes. Using time series of fisheries landings from a global database, their model shows that interannual sea surface temperature variation is integrated through each trophic level of the food web and can underlie observed low-frequency population dynamics.

## Introduction

Many marine fish populations experience conspicuous fluctuations in abundance on multiple-year to decadal time scales^1–3^. These low-frequency fluctuations in fish populations can negatively impact upper-trophic wildlife species such as seabirds^4^, as well as the cultures and economies of coastal societies dependent on fisheries^5^. Despite their significance, the attribution of environmental, demographic, and catch-related drivers of population fluctuations continues to be a major challenge in fisheries science^6,7^. A lack of understanding may limit attempts to forecast fisheries populations, thereby increasing the risks of overestimating sustainable catch quotas^8^. To date, quantitative models of low-frequency variability in marine fish populations remain underdeveloped. Existing population models are commonly species-specific and require high-precision data on fish vital rates, limiting their application in data-poor situations which characterize the majority of our global catch^9^.

While the mechanisms controlling fish population growth are inherently complex, two sets of recent scientific insights aid the development of predictive models of low-frequency dynamics in marine fish populations. First, it is evident that variability in somatic growth of individual fish is often tightly, and linearly, related to variability in environmental conditions, especially sea surface temperature (SST). This relationship is consistent across latitudes and across species of different life spans (see review of literature in Table S1). Second, comparisons among marine fish populations suggest that biomass fluctuations linked to environmental variability may be mediated by life history characteristics such as generation time and trophic level^10–13^. For example, small and short-lived planktivorous fish like sardines and anchovies are more prone to higher-frequency population fluctuations in comparison to large and long-lived piscivorous fish populations such as rock fish (*Sebastes* spp.). We illustrate this point by comparing the magnitude of low-frequency variability in biomass times series of fish species in the North- and Celtic seas (Fig. S1). Shorter generation times due to life history or fishing-induced demographic truncation may be linked to a faster environmental response^11,14,15^. In contrast, a buffering of environmental signals by longer-lived predatory fish may be related to ‘bet-hedging’ strategies of fat storage and flexibility in spawning times that serve to increase individual survival during stressful times and ensure adequate recruitment when favourable conditions arise^16–20^. As a consequence, environmental stochasticity is dampened in populations with a broad age structure (i.e., many mature cohorts), resulting in conspicuous low-frequency variability in their temporal dynamics^14,15^.

Inspired by theoretical work on age-structured populations^10,11^ we build upon these two concepts to present and validate a new modelling approach in which the dynamics of marine organisms are an “integration” of environmental variability over multiple years through demographic processes^12,16^. Specifically, we assess to what extent observed low-frequency variability in fish populations could be attributed to integrated SST variability. Lastly, we use our modelling approach to predict how fish biomass may respond to increasing temperature mean state and variance under a changing global climate.

## Autoregressive modelling

To test the hypothesis that observed fish population dynamics contain signatures of integrated SST variability, we used autoregressive models to simulate this integration or “biological buffering”^12^. For species at the first trophic level (TL1), we calculate the annual anomaly in population biomass *B*_TL1_ at time *t* as:

1 $${B}_{{{{{{\rm{TL1}}}}}}}(t)={{{{{\rm{SST}}}}}}(t)+{\tau }_{{{{{{\rm{bio}}}}}}}({B}_{{{{{{\rm{TL1}}}}}}}(t-1))$$

with SST representing standardized (i.e., converted to *z*-score) interannual SST variability, *B*_TL1_(*t* − 1) the standardized population biomass in the previous year and *τ*_bio_ a factor quantifying how *B*_TL1_(*t* − 1) affects *B*_TL1_(*t*)^12^. The factor *τ*_bio_ is calculated as:

2 $${{\tau }_{{{{{{{\rm{bio}}}}}}}}\;=\;{\displaystyle \frac {{{{{{{\rm{longevity}}}}}}}-1}{{{{{{{\rm{longevity}}}}}}}}}}$$

We chose this formulation because the biomass dynamics generated are strongly dependent on fish longevity. For example, for short-lived species with near annual life cycles, the *τ*_bio_ ≈ 0, causing *B*(*t*) to be independent of *B*(*t* − 1) and *B*(*t*) time series to closely track environmental variability. For longevity <1 year, we set *τ*_bio_ to zero, which is generally the case at the first trophic level. For long-lived species on the other hand, the formulation of *τ*_bio_ induces strong carry-over effects, creating much stronger patterns of low-frequency variability relative to interannual variability.

For trophic levels 2–4, we assume food availably is a major driver of population fluctuations. Thus, for these trophic levels, SST(*t*) is replaced by *B*(*t*) (the standardized population size) of the next-lower trophic level (TL-1), resulting in:

3 $${B}_{{{{{{\rm{TL}}}}}}}(t)={B}_{{{{{{\rm{TL-1}}}}}}}(t)+{\tau }_{{{{{{\rm{bio}}}}}}}({B}_{{{{{{\rm{TL}}}}}}}(t-1))$$

A “species” trophic position determines how many times Eq. (3) is repeated, building on the dynamics of the next lower trophic level. This method thus assumes a bottom-up propagation of environmental signals at higher trophic levels (e.g., through food availability^21^). We do acknowledge potential top-down controls on population growth^22^, but in our simple model make the implicit assumption that bottom-up effects are stronger. It is commonly held that variability in marine populations is caused primarily by recruitment variability^23^ which, regardless of adult trophic position, may relate more directly to environmental conditions and/or plankton availability and thus less so to the next lower trophic level. Although recent studies challenge this paradigm and show that somatic growth can be an important driver of population fluctuations^21,24^, it is likely that our model approach will overestimate low-frequency variability in populations for which recruitment success is an important driver of changes in population size. In such cases, a lower number of integrations (i.e., iterations of Eq. (3)) and/or adjustment of *τ*_bio_, commensurate with the age at which a species’ recruits to the adult population, could be more appropriate. However, for many species there is likely strong collinearity between vital rates, (e.g., high growth years coincide with years of low mortality and high fecundity/recruitment, and vice versa), all driving population growth in the same direction^25^. Hence recruitment fluctuations are implicitly included in our model. Our approach could thus be regarded as a null model that quantifies the maximum level of bottom-up influence through environmental forcing on fish biomass dynamics. We stress that the output of our modelling approach differs from a low-pass filter or a running average which solely emphasise and attenuate properties of the focal time series. Instead, autoregressive models induce phase shifts and amplify low-frequency variability as environmental variability passes through multiple tropic levels.

## Results and discussion

### A North Sea marine food web

**Fig. 1:**
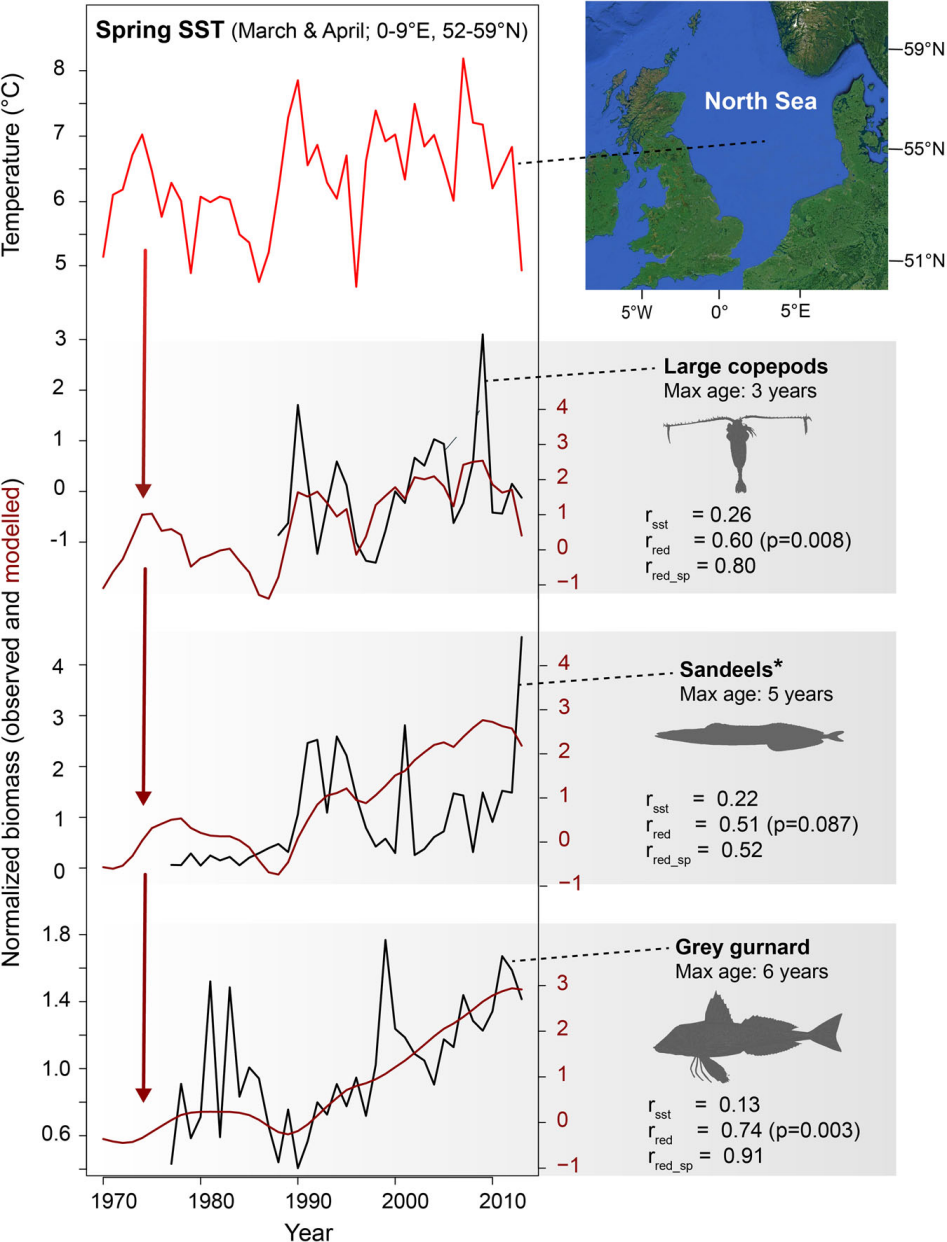
Integration of interannual SST variation corresponds to low-frequency fluctuations in biomass. In this example from the North Sea, autoregressive modelling of high-frequency sea-surface temperature (SST; red lines) generates patterns that match variations in population biomass (black lines) across three trophic levels. Results on the right show correlations at each trophic level between: spring SST data and biomass (*r*_sst_); auto-regressed SST and biomass (*r*_red_); and between a cubic smoothing spline (smoothing parameter: 0.5) through the biomass data and auto-regressed SST (*r*_red_sp_). The latter was included for a more direct comparison of low-frequency variability that may be masked in relatively uncertain biomass assessments. The *p*-values are determined using data simulations (see “Methods”). *Poor association for sandeels could be caused by substantial sampling errors in assessment data^48^. Data used can be found in Supplementary Data files 1 and 2.

Most fish species monitored in stock assessment programmes are commercially important and their population dynamics are likely strongly affected by exploitation. The most common demersal fish species in the North Sea, the grey gurnard, however, has no commercial importance and its abundance has been surveyed consistently since 1977^26^. We illustrate the skill of our approach by modelling the dynamics of grey gurnard and its main prey items (Fig. 1). The autoregressive models closely match the low-frequency variability in observed grey gurnard populations, suggesting that interannual SST variation is integrated through each trophic level of the food web, and that our autoregressive modelling approach can generate realistic decadal-scale fish population dynamics.

### Modelling low-frequency populations dynamics globally

For a global application of our modelling approach we first used a life history database of 3917 fish species^27^ to establish relationships among longevity, temperature, and trophic level (i.e., to parametrize τ_bio_ in Eqs. (1) and (3). We found that within a given trophic position, average fish longevity decreased with mean SST (Fig. S2 and Table S2), which is consistent with previous findings^28,29^. This pattern reflects a known trade-off between metabolism and growth, which both increase with SST, versus maturity and longevity, which both decrease with SST^30–32^. In addition, the observed longevity-temperature relationship is likely also related to the temperature size rule, which predicts an inverse relationship between fish size and mean temperature, and thus a positive relationship between size and latitude^33^.

**Fig. 2:**
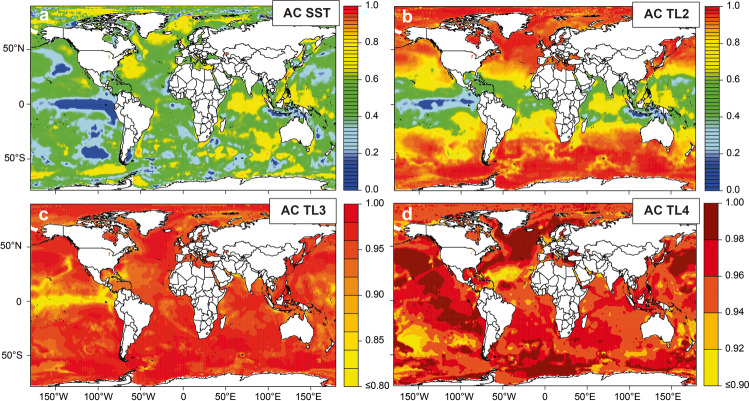
Spatial patterns of first-order autocorrelation (AC) in sea surface temperature (SST) and results of autoregressive models of fish populations at different trophic levels (TL). For all 1 × 1° gridded time series, first-order autocorrelation was calculated over the period 1950–2018. Simulated fish biomass dynamics is based on autoregressive models (Eqs. 1–3), using annual SST time series and the relationship between temperature and fish longevity (Fig. S2 and Table S2).

Next, we developed autoregressive models from the inverse relationship between temperature and longevity (Fig. S2 and Table S2) and gridded SST anomalies (1950–2018) to simulate fish biomass anomalies in each 1° × 1° grid cell across the world’s oceans. This was done separately for trophic levels 2–4, excluding species at trophic level >4, as these include highly migratory species (e.g., tunas, swordfish, and large sharks) that can be more responsive to basin-wide as opposed to regional environmental conditions^34^. We extracted the level of first-order autocorrelation from the simulated biomass time series, where higher values indicate greater low-frequency variability. As expected, autocorrelation increased with trophic level (Fig. 2). Strong latitudinal gradients also appeared, suggesting spatial differences in the fundamental behaviour of populations even at the same trophic level. Spatial patterns were especially apparent in low trophic-level species (TL2; i.e., planktivorous species), and can logically be attributed to greater longevities in cooler climates^28^ as well as increasing SST autocorrelation with latitude (Fig. 2a). For mid and upper trophic levels (TL3-4; i.e., omnivorous and piscivorous species), latitudinal patterns were weaker. We repeated our autoregressive models on gridded data of ocean NPP (2003–2018), which are based on remote sensing data of surface chlorophyll estimates, SST, and PAR^35^. Patterns of increasing autocorrelation with latitude (for low trophic levels) were similar to those found using the SST time series (Fig. S3).

High temporal autocorrelation may indicate slow recovery rates of fish populations after exploitation, climate extremes, or other disturbances^36^. In contrast, populations with low autocorrelation are likely to respond strongly to year-to-year changes in climatic conditions, are therefore less buffered, and thus more sensitive to climate anomalies^37^. In these species, extreme climate conditions may cause sudden population growth or collapse. Our models indicate this is more likely to happen in short-lived and lower-trophic-level fish species, which is in agreement with results from stock-assessment data^38,39^.

### Comparison to fisheries landings

**Fig. 3:**
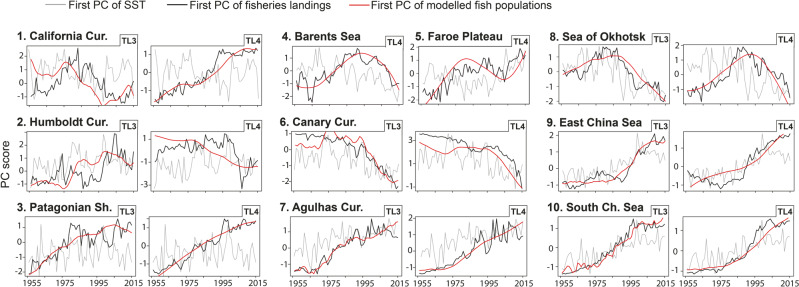
Simulated marine fish dynamics matches independent empirical data from landings. Examples of covariance between simulated (red) and observed (black) fish populations in ten LMEs. Observed data is the first principal component of the catch data within the LME, while modelled data is the first principal component of simulated populations across all 1 × 1° grids within the LME (see Table S3 for variance explained per PC). Trophic levels 3 and 4 (TL3 & TL4) are both shown except for the Barents Sea and Faroe plateaus due to poor data availability. The thin grey line in each figure is the first principal component of SST data in each LME. Numbers correspond to locations in Fig. 4a.

We compared our global simulation of fish population dynamics to fisheries landings (3789 time series spanning 1955–2014) reported by the Food and Agriculture Organization and reanalyzed by Pauly and Zeller^9^. For species at lower trophic levels (TL2), latitudinal gradients in autocorrelation are mirrored in fisheries landings (Fig. S4). Yet for mid and upper trophic levels, we found no significant correlation between simulated and observed mean levels of autocorrelation within large marine ecosystems (LMEs). This lack of correspondence is likely a consequence of the small range of autocorrelation values at these high trophic levels. Nonetheless, for many LMEs, the simulated fish population time series track observed landings remarkably well (Fig. 3). Indeed, we found correlation coefficients of *r* > 0.7 (*r*^2^ ≥ 0.5) between the first principal components of modelled and observed time series in 29, 54, and 62% of the tested LMEs for trophic levels 2, 3, and 4, respectively. Very strong correlation coefficients (*r* > 0.9, *r*^2^ ≥ 0.8) were found in 0, 21 and 29% of the tested LMEs for trophic levels 2, 3 and 4, respectively (Tables S3–5). Running the same analyses, but using random data instead SST in the models, indicated that such high correlations are unlikely caused by chance or an artifact of adding temporal autocorrelation to an explanatory time series (Fig. S5). While long-term trends in SST and fishing intensity may drive some high correlations, in many LMEs biomass turning points are correctly simulated from SST (Fig. 3). This indicates that the integration of environmental signals could be responsible for a significant portion of observed low-frequency variability in fish populations.

**Fig. 4:**
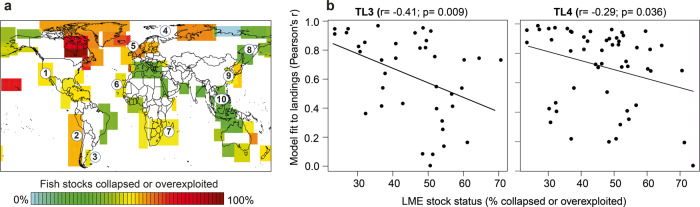
Overfishing can diminish climate-driven fluctuations in fish populations. **a** Stock status, defined as the percentage of stocks collapsed or overexploited over 1950–2010 for all large marine ecosystems (data from^61^). The numbers in the map correspond to the examples given in Fig. 3. **b** Fit of simulated fish biomass dynamics to that observed in landings is highest in LMEs experiencing low exploitation pressure and decreases with stock status, for trophic levels (TL) 3 and 4. Model fit was estimated as the correlation of the first principal component of landing data to the first principal component of simulated fish populations within each LME. Only LMEs with at least 5 time series of landings were included here.

In recent decades, industrialized fishing has put tremendous pressure on the World’s marine ecosystems (Fig. 4a), with strong and lasting effects on fish stocks^40^. We expected the correspondence between simulated and observed population biomass to be relatively low in heavily exploited populations as they have suffered age and size truncation^41,42^ which would reduce temporal autocorrelation and make these relatively long-lived species behave like species with shorter lifespans. Indeed, when comparing the first principal components of simulated fish stocks to observed landings, we found stronger agreement in low-exploitation LMEs than overexploited LMEs (Fig. 4b). Our models do not account for this exploitation effect and could thus overestimate the degree of low-frequency variability in intensively exploited LMEs (Fig. S6).

### Effects of climate change

**Fig. 5:**
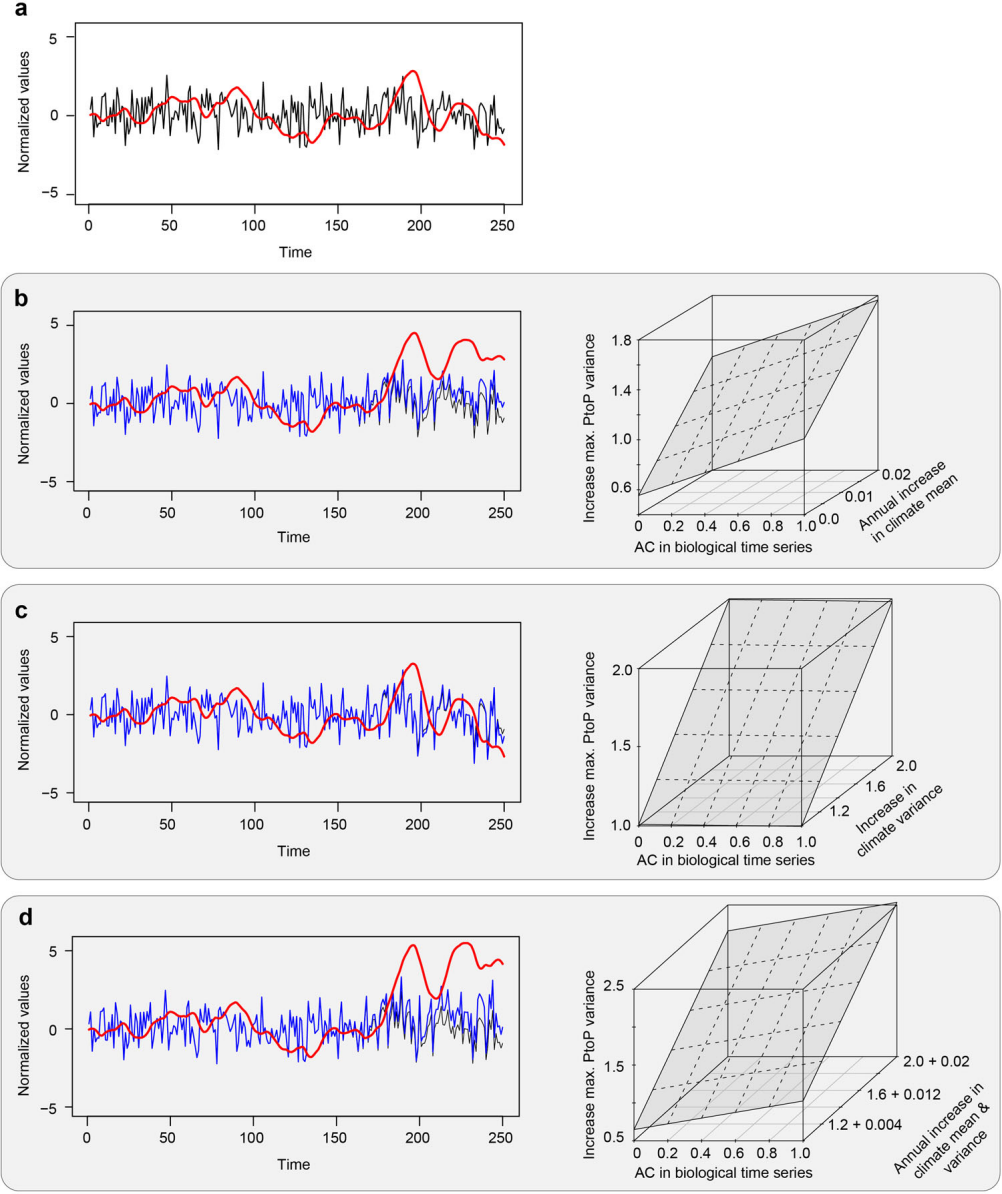
Climate change may amplify marine fish biomass dynamics. **a** We depart from a random time series representing SST variability without changes in climate mean or variability (black line), and the simulated biomass dynamics of a marine fish species at trophic level 3 (red line). For this simple study system, we next apply gradual climate change (blue lines) during a 100-year period by adding an SST warming trend (**b**), an increase in SST variance (**c**), and their combination (**d**). Each of these climate change scenarios was repeated 16,500 times using a range of fish longevities, varying degrees of warming and variance trends, and with varying initial random SST time series (black line in **a**). The results of the simulations are summarized in the right panels. Relative change in maximum peak-to-peak variance (PtoP) pre-trend vs. post-trend was calculated to represent changes in the amplitude of low-frequency variability. PtoP values > 1 indicate an increase in low-frequency amplitude. First-order autocorrelation (AC) in the simulated fish populations before climate change (i.e., from years 1 to 150) is shown on the *x*-axis of the three-dimensional plots.

The future marine climate will likely be characterized by even greater warming and increasing climate variance^43,44^. Using data simulations, we tested how rising trends in SST mean state (Fig. 5b) and/or variance (Fig. 5c, d) might affect the low-frequency dynamics of fish populations. Results from these simulations suggest that an increase of either mean SST state or variance induces stronger fluctuations in population biomass, with the greatest amplitudes occurring when SST mean state and variance increase simultaneously (Fig. 5d). An important caveat is that species may shift their distributions to follow their thermal niche, which may help mitigate some of these impacts^45,46^. Yet, our results suggest that rising SST averages and variability must be considered as sources of increased volatility in fish biomass and associated catch that could lead to greater instability of wildlife populations and coastal economies dependent on marine fish populations.

While we acknowledge the existence and influence of other biological causes of low-frequency variability in biomass, our autoregressive approach offers a simple and broadly applicable null model of climate-induced fish population dynamics. Such information may help disentangle the influence of physical forcing from that of fishing pressure. It also affords some level of predictive power given that biomass is a function of lagged climate phenomena, which is of direct relevance to the management and sustainability of these fish stocks. Moreover, these autoregressive approaches may provide basic predictions of population biomass responses to climate variability and climate change not just for fish, but any upper-trophic species.

## Methods

### North Sea marine food web

The abundance of grey gurnard (*Eutigla gurnardus*) has been consistently measured in ICES research vessel surveys since 1977. Grey gurnards feed primarily on crustaceans as juveniles, increasing their consumption of fish up to 95% of their diet as they grow^47^. Sandeel species (*Ammodytes*, *Gymnammodytes,* and *Hyperoplus*) are the main prey species of grey gurnards >10 cm SL (about 30−50% of the fishes eaten). Sandeels are also surveyed in the North Sea as part of ICES stock assessments (the abundance of six species are grouped together). Sandeels are found across the entire North Sea and are exploited only on the western side of the basin^48^. In turn, the abundance of sandeels has been linked to the abundance of large copepod prey species^49,50^; abundance data of the latter were published by Capuzzo et al.^51^. Thus, the availability of abundance data across three linked trophic levels allowed the testing of autoregressive models (Fig. 1). We used spring sea surface temperature (SST in March and April)^52^ as a potential climate driver of the food chain, as averaged across the North Sea (0–9°E and 52–59°N). SST in spring reflects growing season length and has been reported to affect the abundance of copepods^53^, as well as the period of highest growth for sandeels^54,55^. Autocorrelation was added to SST in our models based on species longevity with a maximum age of 6–8 years for grey gurnard^56^, 4–10 years for sandeels^48,57^, and 1–4 years for large copepods. Testing for significance: One issue with high levels of autocorrelation is that statistical assumptions of serial independence are violated, complicating efforts to establish the significance of correlations or regressions^58^. To address this problem, we apply an alternative significance test based on data simulations^59^. For the grey gurnard case study, we created 100,000 random (i.e., artificial) “environmental” time series with the same length, mean, standard deviation, and autocorrelation as the environmental data (spring SST). We correlated each of these random “environmental” time series to the biological time series studied (e.g., large copepods, sandeels and grey gurnard). Those random “environmental” series that correlated as strongly to the biological time series as the original ‘real’ SST data were retained (i.e., same Pearson *r* ± 0.05). Next, these retained “environmental” random time series were reddened (i.e., autocorrelation added), using autoregression models parameterized with species’ longevities, and we recorded the extent to which the correlation with the biological time series changed. The goal here was to estimate the increase in correlation coefficient in random, unrelated time series, simply by adding low-frequency variability. We then compared this distribution of “baseline” correlation changes from the simulated data to the change in correlation when autocorrelation was added to instrumental SST. We deemed a relationship significant if the correlation increase for the observed SST data was unusually high (>95th percentile) relative to the ensemble of random “SST” data.

### Assessing spatial patterns in fish longevity

The relationships among ocean temperature, trophic position, and fish maximum age were analyzed using data of 3917 marine fish species from 21 LMEs (Fig. S2). These LMEs were chosen randomly, but as such that they spanned a gradient from low- to high temperature regions. For each LME, life-history trait data were retrieved from the online databank Fishbase^27^. For each marine ecosystem, mean annual sea surface temperature from 1950 to 2018 was calculated over an central area at least 1° × 1° in extent using the Hadley Centre Sea Surface Temperature dataset^52^. Mean annual SST in these ecosystems ranged from ~0 to 28 °C. We used linear regressions to quantify relationships among temperature, trophic position, and fish maximum age (Fig. S2; Table S2). A discussion of the robustness of this relationship in provided in the supplementary information (under Fig. S2).

### Applying autoregressive models to gridded SST and NPP

Autoregressive models were applied to 1 × 1° gridded SST from 1950 to 2018 of the Hadley Centre Sea Surface Temperature dataset^52^ (Fig. 2). At the lowest trophic position, the environmental driver (*E*) in the model was set as the time series of regional sea surface temperature from 1950 to 2018 (see main text for model description). Autocorrelation was added to the SST time series using a *τ*_bio_ parametrized based on the relationship between mean annual temperature (of the grid cell from 1950 to 2018) and fish longevity (Fig. S2 and Table S2). In the same way, additional autocorrelation was added to this time series to model the next trophic level. These models simulated variability in fish populations for a certain region, given (i) variability of the physical environment (SST), including the level of autocorrelation already present in that physical environment (Fig. 2a), and (ii) the expected longevity of an “average” fish at a certain trophic position (Fig. S2). We repeated the analyses, using gridded estimates of ocean net primary productivity instead of SST (Fig. S3). Gridded NPP was based on the Vertically Generalized Production Model (VGPM) of Behrenfeld and Falkowski^35^, MODIS surface chlorophyll concentrations (Chlsat), MODIS 4-micron sea surface temperature data (SST4), and MODIS cloud-corrected incident daily photosynthetically active radiation (PAR). Monthly gridded data from 2003 to 2018 were obtained from Oregon State University’s Ocean Productivity (https://www.science.oregonstate.edu/ocean.productivity/). We averaged higher-resolution data (1080* 2160 grids) to a 1° × 1° gridded global database. Our model approach was initially inhibited by the length of the NPP data series, spanning only 16 years. We, therefore, repeated the NPP time series in each grid cell once, so that it yielded a 32 year sequence (i.e., 2003–2018 + 2003–2018). Such stitching of two identical time series in each grid cell allowed the models the required time to build up low-frequency variability, but created flawed model output. However, the goal here was not to model the precise low-frequency pattern exhibited by fish populations, but to examine if general autocorrelation levels, and in particular their spatial patterns, are comparable to the analyses based on SST data (1950–2018).

### Autocorrelation in FAO global landings

Fisheries landings recorded by the Food and Agriculture Organization (FAO) of the United Nations were used to verify model output. Catch data were organized into 65 LMEs^9^. We used all data available, including marine finfish and invertebrates, but filtered data according to the following conditions: (i) time period spanning 1955–2014 (60 years), (ii) less than 25% missing values, and (iii) having species-level identification (removing unidentified and miscellaneous groups). This yielded 3789 species time series, with at least 55 years of data for each species. No time series were further removed. Within each LME, the average autocorrelation was calculated across all fisheries landing time series within each trophic level. Next, we calculated the first principal component of all landings within each trophic level of each LME (if *n* > 5 time series), as well as the first principal component of the simulated fish populations (i.e., autoregression models per 1° grid cell based on SST) within each trophic level of each LME. We then compared the leading principal component of landings with the leading principal component of simulated fish populations as a way of broadly assessing the fit between predicted and observed landings (Figs. 3, 4). The sign of the PC of simulated data was reversed in upwelling areas, given the strong relationship between upwelling intensity (usually negatively related to sea surface temperatures) and productivity in these regions^60^.

### Potential effects of climate change

We used data simulations to assess the potential responses of fish populations to changes in climate mean, variance, and their combination. We started each simulation with a random time series of 250 years, normalized to a mean of zero and a standard deviation of one. A theoretical fish population at trophic level 3 was simulated by integrating this random data across a number of longevity scenarios (Fig. 5a), ranging from 1 to 2 years for trophic level 1, 1–3 years for trophic level 2, and 1–5 years for trophic level 3; yielding 30 possible age combinations. Next, we used the original random time series, but added an increasing linear trend ranging from 0.002 to 0.02 (in steps of 0.002) standard deviation per year over the last 100 years for a total of 11 scenarios (Fig. 5b). This approach was repeated 16,500 times, using all possible combinations between the longevity scenarios (*n* = 30), rates of climate change (none + 10 different trends), and random climate time series (50 per longevity × climate change combination). For each simulation, we measured the change in the amplitude of fluctuations over last 100 years between fish populations simulated from the control and the climate change scenarios. This change in amplitude was defined as the maximum peak-to-peak variance in the fish population simulated under a climate change scenario divided by the maximum peak-to-peak variance in fish population simulated under the control scenario. The same approach was also used to simulate the effect of increasing climate variance (Fig. 5c). To this end, we multiplied the last 100 years of the random time series by a factor 1 (no change) to 2 (a doubling of variance), with steps of 0.1 for a total of 11 scenarios. We did not simulate a gradual increase of climate variance, but induced a consistent change across the entire last 100 years of the simulate data. Lastly, we combined increasing mean and variance (Fig. 5d). We did not use all possible combinations, but instead merged each level of the 11 scenarios in mean state with its respective level of the 11 scenarios of change in variance. The resulting 11 scenarios range from no change in mean and variance to a maximum increase in mean of 0.02 standard deviation per year over the last 100 years in combination with the maximum consistent two-fold increase in variance during the last 100 years.

### Statistics and reproducibility

Details about the statistical analyses performed in this study are given in the respective sections of results and methods. The R-scripts used for model simulations are included in Supplementary Data file 5.

### Reporting summary

Further information on research design is available in the Nature Research Reporting Summary linked to this article.

## Supplementary information

Supplementary Information

Description of Additional Supplementary Files

Supplementary Data 1

Supplementary Data 2

Supplementary Data 3

Supplementary Data 4

Supplementary Data 5

Reporting Summary

## Data Availability

The sea surface temperature data used in this study was obtained from the Met Office Hadley Centre (https://www.metoffice.gov.uk/hadobs/hadisst/). Monthly gridded data of marine NPP was obtained from Oregon State University’s Ocean Productivity database (https://www.science.oregonstate.edu/ocean.productivity/). FAO catch data for the period 1955−2014 was obtain using https://rpubs.com/joyceongjl/catchAR1. All other data used are included in Supplementary Data files 1–4.

## Code availability

The R-scripts used for the model simulations (e.g., Figs. 1, 2, and 5) are included in Supplementary Data file 5. All other r-scripts used (e.g., for making the figures) are available upon request.
