# Supplementary material for: Interannual temperature variability is a principal driver of low-frequency fluctuations in marine fish populations
Source: Commun Biol. 2022 Jan 11;5:28. doi: 10.1038/s42003-021-02960-y (PMC8752724; doi:10.1038/s42003-021-02960-y)
Supplement: Supplementary file 1 — Supplementary Information [file 42003_2021_2960_MOESM1_ESM.pdf]

SUPPLEMENTARY FIGURES 1-6

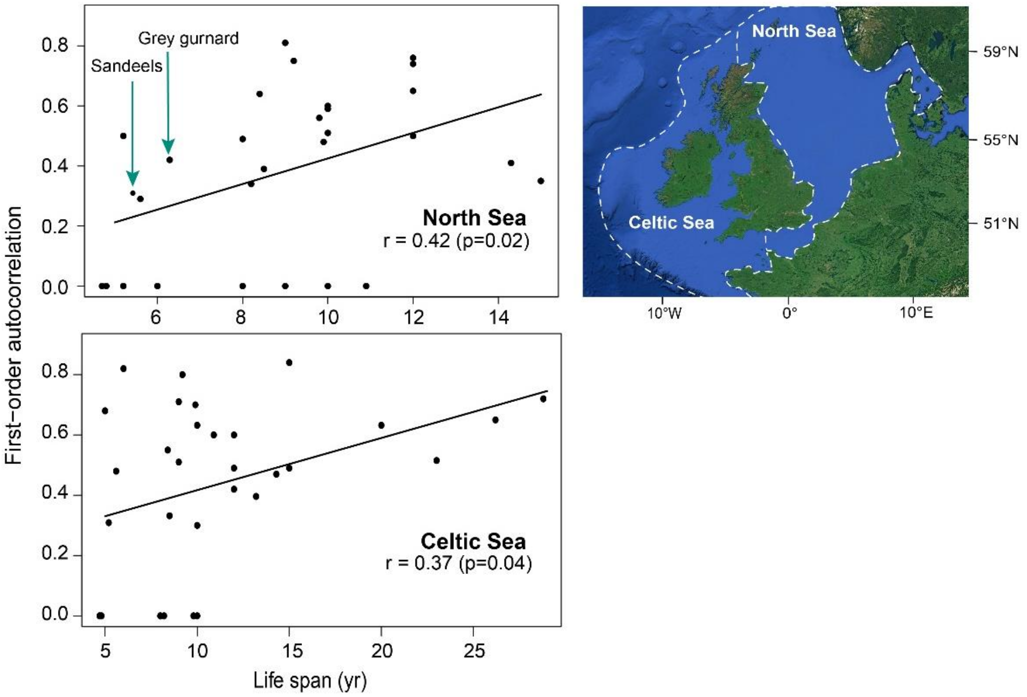

Figure S1 | Temporal autocorrelation in biomass estimates increases with fish maximum age in the North Sea and Celtic Sea. First-order autocorrelation (i.e. lag 1) was determined in ICES abundance estimates, as recently compiled and standardized by Heessen, et al. [1] for the North and Celtic seas. We initially selected all species for which the North Sea and/or Celtic Sea represent a central part of their distributional range (i.e. no species that are only occasionally found in these areas) and for which no known issues with the assessment data where reported in Heessen, et al. [1]. This yielded biomass time series (most covering period 1984-2013) for 31 species in the Celtic Sea and 31 (most covering 1977-2013) species in the North Sea. However, for the North Sea, we excluded Atlantic cod (*Gadus morhua*) due to fishing-induced stock collapses in the time period analysed, and witch (*Glyptocephalus cynoglossus*) because of uncertainty in stock-specific longevity. For some species, the time period over which autocorrelation was calculated was shortened because of very low abundance in the first part of the time series (i.e. a period without temporal variability). Data on life-history traits were acquired from Heessen, et al. [1] and references therein, or from Froese and Pauly [2]. When data of the two regions are combined: r=0.38, p=0.003. Effect of trophic level p=0.08 for the North Sea, and p=0.12 for the Celtic Sea are not shown. The data shown in the figure is included in Supplementary Data 3.

Part of the uncertainty around the temporal autocorrelation – life span relationships is likely due to the challenge of computing autocorrelation from times series of abundance estimates (e.g. because of measurement errors and time series length). The zero autocorrelation observed for some species could have at least three causes: (1) the temporal dynamics of a species' abundance may exhibit very little low-frequency variability and thus lack detectable temporal autocorrelation; (2) zero autocorrelation may reflect the potentially large sampling error in the stock assessment data for some species, which can create large "random" year-to-year fluctuation in the data that conceal low-frequency variability, and (3) the relatively short time span of the stock assessment data could

also inhibit the detection of temporal autocorrelation. Also note that we set non-significant autocorrelations to a value of zero in these analyses and in Figure S1. This explains the lack of data points between zero and values of around 0.3. Arrows in top panel indicated fish species shown in Fig. 1.

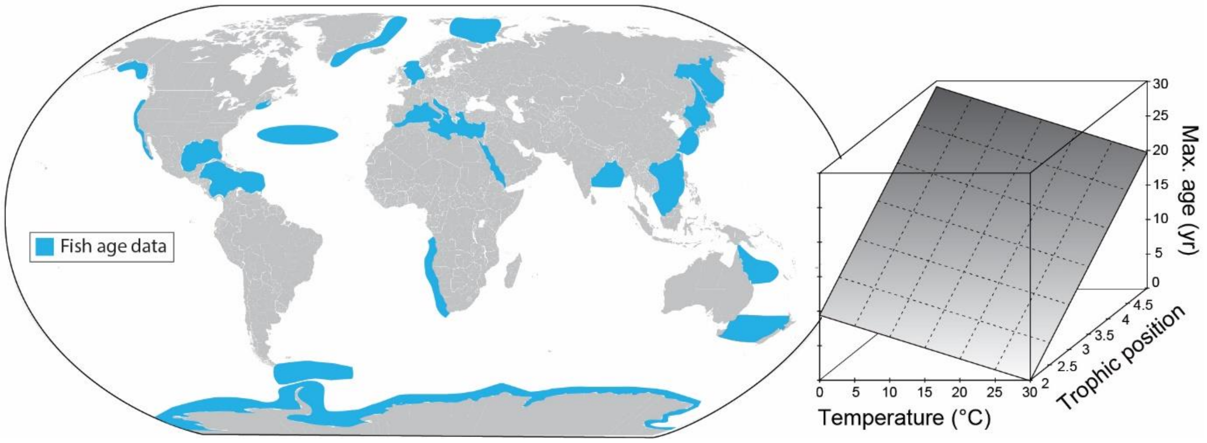

Figure S2 | Determinants of maximum age of marine fish species. Using estimates of the longevity of 3917 marine fish species from 21 large marine ecosystems (areas indicated in left panel), we assessed the relationship between fish maximum age, sea surface temperature and trophic position (data used is included in Supplementary Data 4). The relationship derived from a multiple regression is given in right panel; see Table S2 for model details. The fish maximum age data are largely modelled estimates (e.g. based on the work of Taylor [3]), with about 10% consisting of empirical data[2]. We tested to what extent the relationships shown here and in Table S2, would change when using only empirical data. We used the R package *Rfishbase* to extract empirical data on fish maximum age from the Fishbase database. This yielded results for 573 species globally; for each the source of the age information was available. We found that the correlation between modelled estimates and empirical observations of maximum age was significant ($r=0.51$, $p<0.0001$), and that a multiple regression using empirical data only provided similar results as those found with the larger dataset that also includes modelled estimates of maximum age. Although the estimate of the SST effect on fish maximum age was similar (-0.32 versus -0.24, when using the full dataset or empirical dataset, respectively), the estimated effect of trophic level dropped from 6.59 to 2.76 in the regression based on empirical data only. Next, we re-ran all analyses using the relationship between fish age, trophic level and SST based on empirical data only. We found that the results presented in our manuscript (Figs. 2-4) are almost identical to those using the age relationships derived from the original dataset, with the exception of the spatial patterns found for trophic level 2 (Fig. 2b). A clear latitudinal increase in first-order autocorrelation disappears when using the age-TL-SST relationship based on empirical data only. However, closer inspection of the empirical dataset used, showed that age estimates are virtually absent for fish <30 cm. Thus, the lack of age data for small fish, which constitute the majority of species at low trophic levels and at low latitudes, likely resulted in an overestimation of the max age of fishes in tropical waters and at low trophic levels and hence explains the much lower effect of trophic level on maximum age and the change in the spatial pattern at TL 2. This interpretation is corroborated by the finding that the presence of a latitudinal change in first-order autocorrelation is supported to some extent by observational data (i.e. autocorrelation in timeseries of landings;

Fig. S4). We therefore used the relationship between maximum age, SST and TL based on the dataset that also includes modelled data on maximum age in our analyses, given the restricted availability of empirical data on maximum age (in particular for small fish species).

Although we acknowledge there may be substantial uncertainty in the relationship presented in Fig S2 and Table S2, we stress that our goal was not to derive accurate estimates of fish age at a species level, which may be highly uncertain for many species. Instead, our main goal here was to derive an estimate of mean fish longevity at each trophic level per large marine ecosystem (LME), with each LME containing hundreds of species. Mean longevity estimates were subsequently used to parameterize autoregressive models, allowing the comparison of model predictions to the general patterns observed in fisheries landings at each LME (Fig. 3,4).

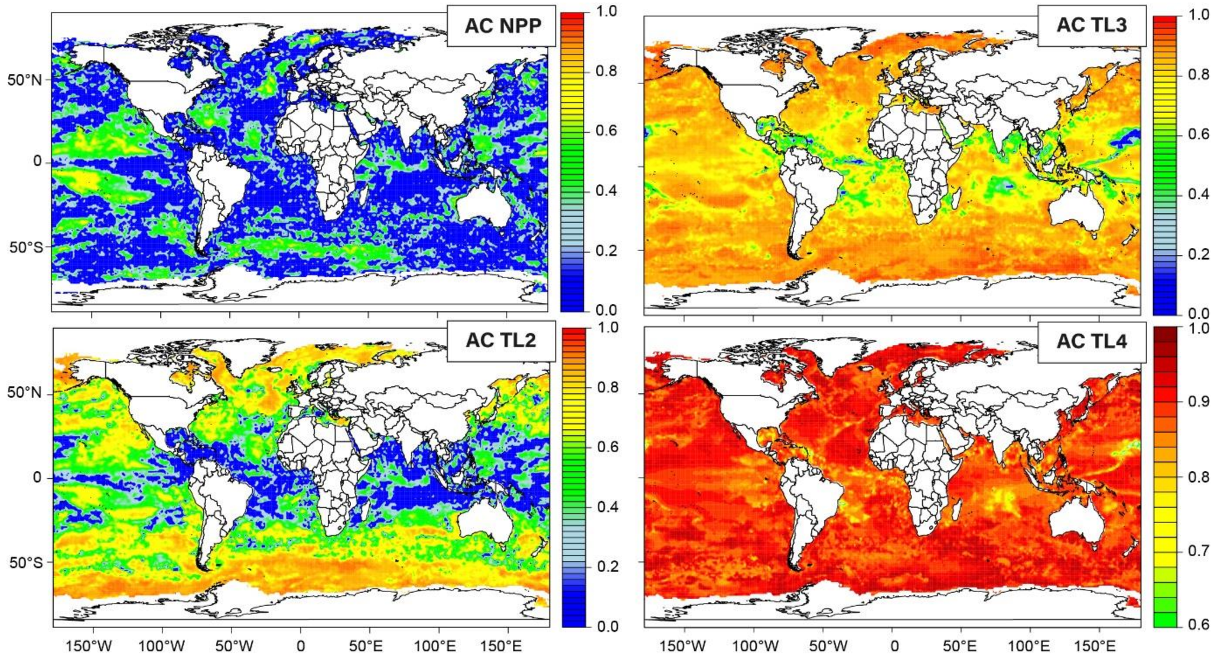

Figure S3 | Spatial patterns of first-order autocorrelation (AC) in ocean net primary productivity (NPP) and simulated fish populations at different trophic levels (TL). Gridded NPP data from 2003-2018 were used to simulate the temporal population dynamics using the attributes of an "average" fish at trophic position 2-4. Autoregressive models were based on annual NPP, and parameterized using the relationships among trophic level, temperature, and fish maximum age (Fig. S2; Table S2). Autocorrelation in these analyses is likely lower compared to those presented in Figure 2 because of the considerably shorter time span of the data used (≤32 years).

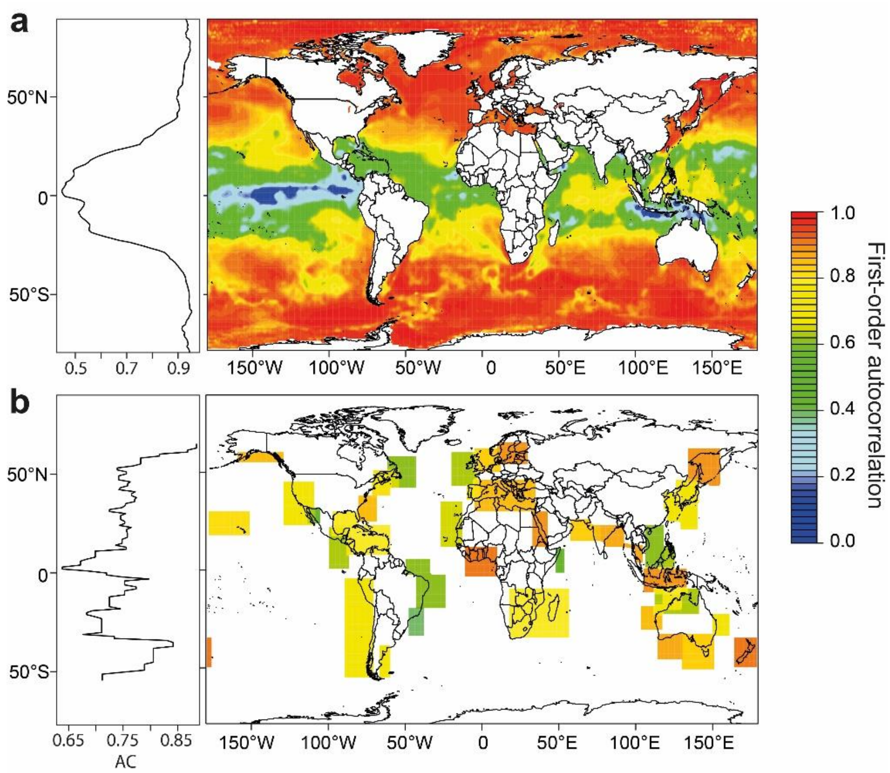

Figure S4 | Comparing temporal autocorrelation in simulated fish and global fisheries landings. Mean first-order autocorrelation in simulated fish populations at trophic level 2 from 1950 to 2018 (**a**) and in fisheries landings from 1955-2014 across large marine ecosystems (**b**). Left panels show mean first-order autocorrelation (AC) per latitude. For simulated fish populations, Pearson correlation between mean first-order autocorrelation and latitude: $r=0.87$, $p<0.0001$. For fisheries landings: $r=0.59$, $p<0.0001$.

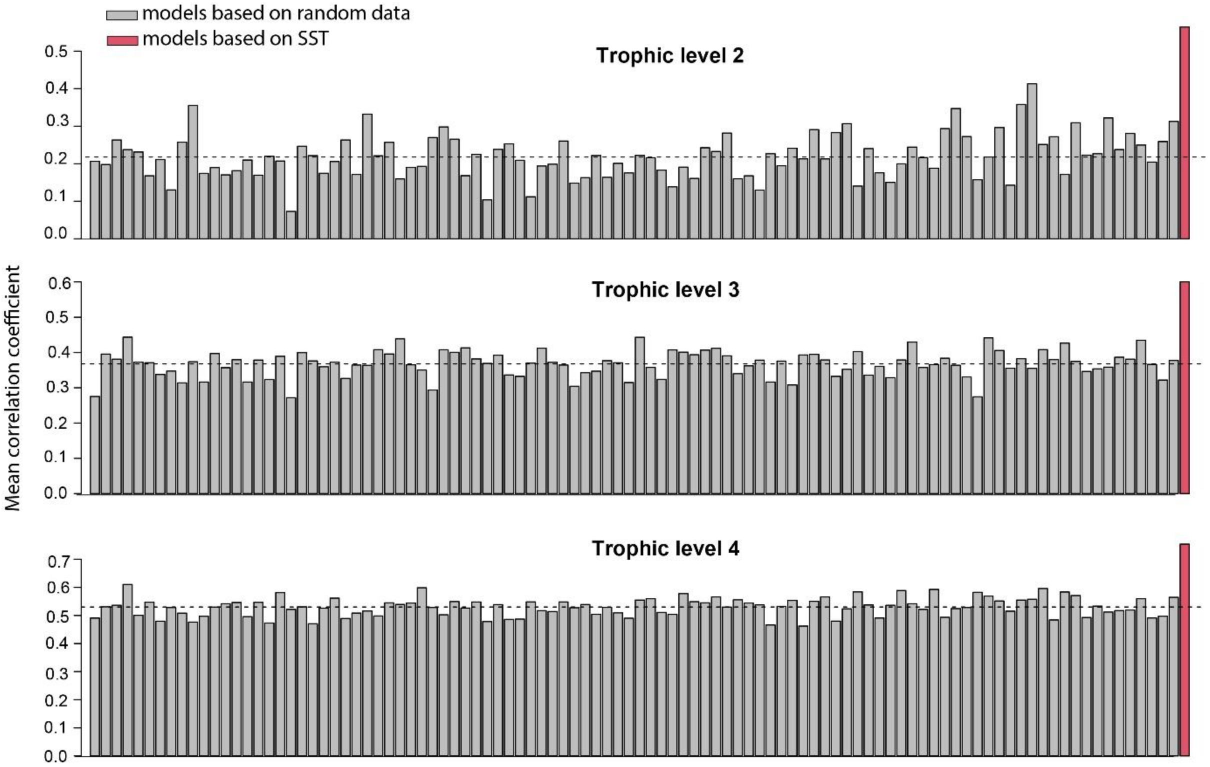

Figure S5 | Correlation coefficients between SST-based models and observations surpass those expected by change. We reran our global analyses 100 times using random data (grey bars) instead of SST (red bar) in our models. In all cases, the first principal component of the observational data (i.e. landings) in an LME was correlated to the first principal component of simulated populations across all 1° grids within the same LME (using random noise or SST). Next, the mean absolute correlation coefficient across all LMEs was taken for each trophic level and shown in the figure. The dashed line shows the overall mean correlation across all 100 runs with random data. Note that for each trophic level, the models using SST data instead of random data have overall correlations that are well above those that could be expected by chance (i.e. the grey bars).

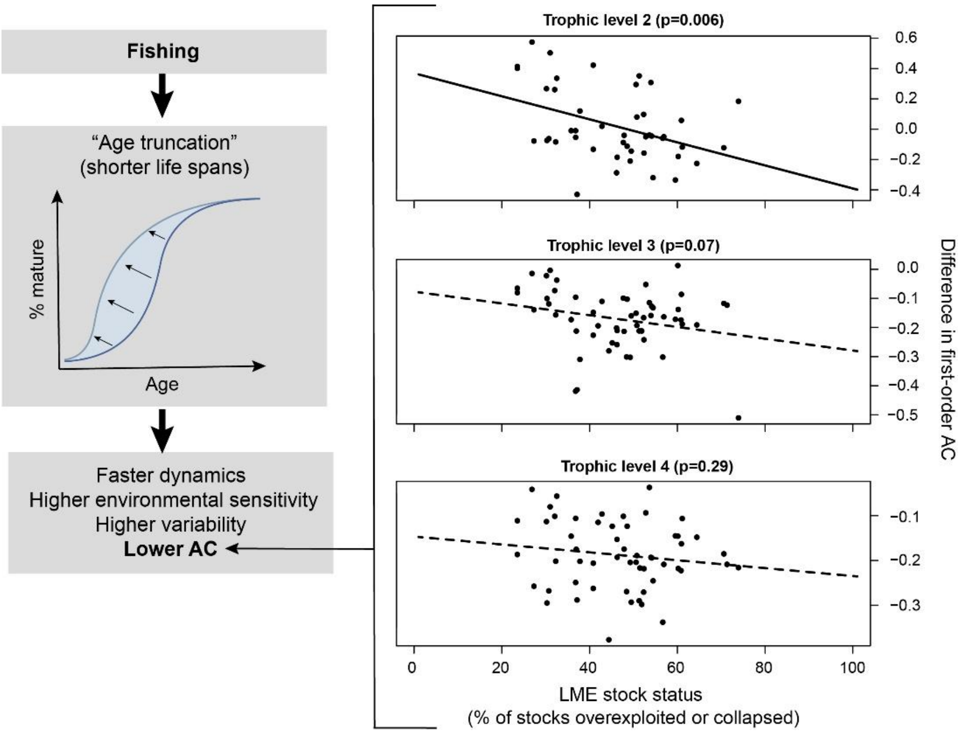

Figure S6 | Relationship between stock status and the difference in mean first-order autocorrelation (AC) of model predictions and of landings for each LME (observed - predicted). At higher level of exploitation, time series of landings generally contain a lower level of first-order autocorrelation than expected based on our model approach (but only significant at TL2). Left panel illustrates how fishing-induced age/size truncation may explain this divergence.

SUPPLEMENTARY TABLES 1-5

Table S1. Review of published fish growth chronologies (based on increment rings in fish otoliths) and the main climate driver(s). These growth chronologies reveal extraordinarily strong correlations of individual fish growth to sea surface temperature (SST) across latitudes. The SST effect indicates the sign of the correlation with growth. Map in top row shows location of studies, and if temperature effects on growth were found.

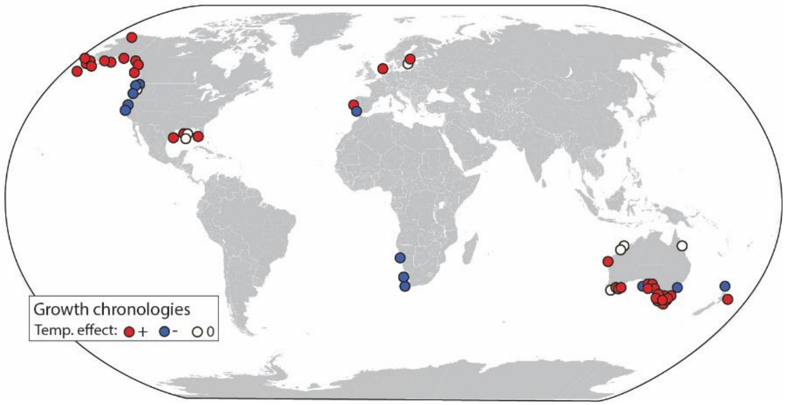

Species	Region	Site(s)	Interval	Lat	Lon	Climate driver identified	SST effect	Citation
Achoerodus gouldii	E Indian Ocean	W Australia	1952-2003	-34.5	120.5	SST, but common variance among individuals was low.	+	Rountrey, et al. [4]
Achoerodus gouldii	E Indian Ocean	SW Australia	1952-2003	-34.3	120.0	SST, but common variance among individuals was low.	+	Rountrey, et al. [4]
Centroberyx affinis	Southwest Pacific	Eastern Bass Strait	1949-1993	-39.2	149	SST	+	Thresher, et al. [5]
Cheilodactylus spectabilis	Southwest Pacific	East Tasmania	1910-2000	-41.8	148.2	SST	+	Neuheimer, et al. [6]
Cheilodactylus spectabilis	Southwest Pacific	Northeast Tasmania	1924-1998	-41.1	148.2	SST	+	Neuheimer, et al. [6]
Cheilodactylus spectabilis	Southwest Pacific	Southeast Tasmania	1910-2000	-43.1	147.9	SST	+	Neuheimer, et al. [6]
Cheilodactylus spectabilis	Southwest Pacific	New Zealand	1932-2000	-35.5	174.7	SST	-	Neuheimer, et al. [6]

Cheilodactylus spectabilis	Southwest Pacific	Victoria	1952-1994	-38	148.5	SST	+	Neuheimer, et al. [6]
Chrysophrys auratus	Southern ocean	Northern Gulf St Vincent	1980-2016	-33.6	137.5	SST	+	Martino, et al. [7]
Chrysophrys auratus	Southern ocean	North Spencer Gulf	1979-2016	-34.7	138	SST	+	Martino, et al. [7]
Chrysophrys auratus	Southern ocean	Southeast South Australia	1983-2014	-37	139.5	SST	+	Martino, et al. [7]
Chrysophrys auratus	Southern ocean	Western South Australia	1992-2016	-32.45	133.2	SST	+	Martino, et al. [7]
Clupea harengus	North Atlantic	Baltic Sea	1946-2016	55.00	17.5	Precipitation and Baltic Sea Index		Smolinski [8]
Helicolenus percoides	South Australia	Kangaroo Island, SA	1994-2012	-36	137	Interaction between MJOindex140E and bottom temperature, resulting in a negative effect on growth.	-	Grammer [9]
Helicolenus percoides	South Australia	Bonney Coast, SA	1993-2012	-38	139.5	Interaction between MJOindex140E and bottom temperature, resulting in a negative effect on growth.	-	Grammer [9]
Helicolenus percoides	southwest Pacific	Southeastern NSW	1975-2011	-37	150	Interaction between MJOindex140E and bottom temperature, resulting in a negative effect on growth.	-	Grammer [9]
Helicolenus barathri	southwest Pacific	Southeastern NSW	1955-2011	-37	150	Interaction between MJOindex140E and bottom temperature, resulting in a negative effect on growth.	-	Grammer [9]
Helicolenus spp.	New Zealand	West NZ	1980-2010	-42	169.5	Interaction between MJOindex140E and bottom temperature, resulting in a negative effect on growth.	-	Grammer [9]
Helicolenus spp.	New Zealand	Chatham Rise NZ	1969-2012	-44	176	Interaction between MJOindex140E and bottom temperature, resulting in a negative effect on growth.	-	Grammer [9]
Hoplostethus atlanticus	Southwest Pacific	Tasmania	1871-1976	-41	148.4	Temperature at 1000m	+	Thresher, et al. [5]
Girella tricuspidata	New Zealand	North Island	1989-2006	-38.5	178.5	SST	+	Gillanders, et al. [10]
Hexagrammos decagrammus	NE Pacific	Katmai	1995-2010	58.4	-153.9	SST	+	von Biela, et al. [11]
Hexagrammos decagrammus	NE Pacific	Prince William Sound	1995-2011	60.1	-147.6	SST	+	von Biela, et al. [11]
Hexagrammos decagrammus	NE Pacific	Elfin Cove	1995-2012	58.2	-136.5	SST	+	von Biela, et al. [11]
Hexagrammos decagrammus	NE Pacific	Whale Bay	1995-2013	56.7	-135.2	SST	+	von Biela, et al. [11]
Hexagrammos decagrammus	NE Pacific	Nuchatlitz	1995-2014	49.9	-127.3	None		von Biela, et al. [11]
Hexagrammos decagrammus	NE Pacific	Clayoquot	1995-2015	49.0	-125.9	None		von Biela, et al. [11]
Hexagrammos decagrammus	NE Pacific	Neah Bay	2000-2010	47.9	-124.9	None		von Biela, et al. [11]

Hexagrammos decagrammus	NE Pacific	Big Sur	2000-2011	36.0	-121.7	None		von Biela, et al. [11]
Lepidopsetta polyxystra	Bering Sea	southeastern Bering Sea	1989-2006	57.5	-164.0	Summertime Sea bottom temperatures	+	Matta, et al. [12]
Lethrinus nebulosus	E Indian Ocean	W Australia	1984-2003	-24.0	113.0	Rainfall and SST	+	Ong, et al. [13]
Leviprora inops	E Indian Ocean	SW Australia	1994-2007	-35.0	118.0	Mean SST during austral summer	+	Coulson, et al. [14]
Limanda aspera	Bering Sea	southeastern Bering Sea	1964-2006	57.0	-163.0	Summertime Sea bottom temperatures	+	Black, et al. [15]
Limanda aspera	Bering Sea	Bering Strait	1984-2008	64.0	-169.0	Bottom temperature	+	Matta, et al. [16]
Limanda aspera	Bering Sea	St. Matthew Island	1988-2008	61.5	-169.0	Bottom temperature	+	Matta, et al. [16]
Lutjanus argentimaculatus	E Indian Ocean	NW Australia	1975-2003	-19.0	119.0	Niño-4/ La Nina	-	Ong, et al. [17]
Lutjanus bohar	E Indian Ocean	NW Australia	1958-2007	-16.5	121.0	Summer SLP / PDO		Ong, et al. [18]
Lutjanus bohar	E Indian Ocean	NW Australia	1952-2000	-18.0	147.0	Winter SLP / Dipole Mode index		Ong, et al. [18]
Lutjanus campechanus	N Gulf of Mexico	Louisiana coast	1975-2003	28.0	-90.0	Winds in March		Black, et al. [19]
Lutjanus campechanus	N Gulf of Mexico	Texas coast	1984-2004	27.5	-96.5	Spring SST, wind stress, and SLP	+	Dzaugis, et al. [20]
Lutjanus griseus	N Gulf of Mexico	Florida coast	1974-2006	28.0	-84.5	Winds and SST in March and April	+	Black, et al. [19]
Nemadactylus macropterus	Southwest Pacific	Eastern Bass Strait	1954-1992	-39.2	149	SST	+	Thresher, et al. [5]
Merluccius merluccius	Northeast Atlantic	Portugal	1973-2015	39	-9	Age-dependent winter and spring SST	-	Vieira, et al. [21]
Merluccius paradoxus	Southeast Atlantic	Namibia	1982-2013	-27	14	Positively related to winter upwelling and negatively correlated to spring SST	-	Wilhelm, et al. [22]
Platichthys flesus	North Atlantic	Baltic Sea	1942-2015	55.00	17	Autumn Baltic Sea Index and Spring SST	+	Smolinski and Mirny [23]
Platycephalus laevigatus	southwest Pacific	Victoria, Australia	1982-2014	-38.8	146.3	Summer-Autumn SST and Spring-Summer freshwater inflows	+	Barrow, et al. [24]
Platycephalus laevigatus	E Indian Ocean	SW Australia	1992-2007	-35.0	118.0	Mean SST during austral summer	+	Coulson, et al. [14]
Platycephalus richardsoni	southwest Pacific	Newcastle, Australia	2001-2006	-33	152.2	SST	-	Morrongiello and Thresher [25]
Platycephalus richardsoni	southwest Pacific	NSW	1981-2009	-35.4	150.9	SST	+	Morrongiello and Thresher [25]
Platycephalus richardsoni	southwest Pacific	Western Bass Strait	1996-2008	-37.9	141.3	SST	+	Morrongiello and Thresher [25]
Platycephalus richardsoni	southwest Pacific	Eastern Bass Strait	1972-2009	-39.2	149	SST	+	Morrongiello and Thresher [25]
Platycephalus richardsoni	southwest Pacific	Central Bass Strait	1997-2009	-39.6	145.8	SST	+	Morrongiello and Thresher [25]
Platycephalus richardsoni	southwest Pacific	Eastern Tasmania	1980-2009	-42.3	147.4	SST	+	Morrongiello and Thresher [25]

Platycephalus richardsoni	southwest Pacific	Western Tasmania	1982-2008	-42	145.1	SST	+	Morrongiello and Thresher [25]
Pleuronectes platessa	North Atlantic	North Sea	1985-2014	56	4	Autumn bottom temperature	+ and – (depending on age)	van der Sleen, et al. [26]
Pleuronectes quadrituberculatus	Bering Sea	southeastern Bering Sea	1987-2006	57.0	-166.0	Summertime eastern Bering Sea bottom temperatures	+	Matta, et al. [12]
Pogonia cromis	N Gulf of Mexico	Louisiana coast	1964-2006	29.0	-92.0	Spring SST, wind stress, and SLP	+	Dzaugis, et al. [20]
Polyprion oxygeneios	E Indian Ocean	SW Australia	1990-1998	115.2	-34.9	Strength of the Leeuwin Current		Nguyen, et al. [27]
Scomberomorus cavalla	Gulf of Mexico	Gulf of Mexico	1986-2010	26.0	-90.0	Atlantic Multidecadal Oscillation		Dzaugis, et al. [20]
Sebastes alutus	Bering Sea	central Bering Sea	1929-2006	56.0	-171.0	SST	+	van der Sleen, et al. [28]
Sebastes aurora	NE Pacific	Oregon coast	1980-2004	44.0	-124.5	Not examined		Thompson and Hannah [29]
Sebastes diploproa	NE Pacific	California coast	1948-2006	39.0	-124.0	Winter upwelling	-	Black, et al. [30]
Sebastes melanops	NE Pacific	Alaska Peninsula	1985-2010	55.1	-161.3	SST	+	von Biela, et al. [11]
Sebastes melanops	NE Pacific	Katmai	1990-2010	58.4	-153.9	SST	+	von Biela, et al. [11]
Sebastes melanops	NE Pacific	Prince William Sound	1995-2010	60.1	-147.6	SST	+	von Biela, et al. [11]
Sebastes melanops	NE Pacific	Elfin Cove	1995-2011	58.2	-136.5	SST	+	von Biela, et al. [11]
Sebastes melanops	NE Pacific	Whale Bay	1995-2012	56.7	-135.2	SST	+	von Biela, et al. [11]
Sebastes melanops	NE Pacific	Nuchatlitz	1995-2013	49.9	-127.3	SST	-	von Biela, et al. [11]
Sebastes melanops	NE Pacific	Clayoquot	1995-2014	49.0	-125.9	SST	-	von Biela, et al. [11]
Sebastes melanops	NE Pacific	Neah Bay	2000-2010	47.9	-124.9	SST	-	von Biela, et al. [11]
Sebastes melanops	NE Pacific	Big Sur	2000-2011	36.0	-121.7	SST	-	von Biela, et al. [11]
Sebastes ruberrimus	NE Pacific	California coast	1950-2003	39.0	-124.0	Indices of cool ocean conditions	-	Black, et al. [31]
Sebastes ruberrimus	NE Pacific	Triangle Island	1954-2003	50.9	-128.7	Warm ocean conditions, especially from the prior summer through the spring of the current year	+	Black, et al. [31]
Sebastes ruberrimus	NE Pacific	Bowie Seamount	1961-1998	53.3	-135.6	Warm ocean conditions, especially from the prior summer through the spring of the current year	+	Black, et al. [31]
Sillaginodes punctatus	Southern Australia	South Australia	1985-2010	-35.4	137.1	Winter SST	-	Mazloumi, et al. [32]
Trachurus trachurus	Northeast Atlantic	Portugal	1963-2015	39	-9	East Atlantic pattern, NAO and spring SST	+	Tanner, et al. [33]

Table S2. Regression model for relationship between temperature, trophic position, and the maximum age of fish. AIC of this model (70367) was lower than that of models with only SST (99594) or only trophic level (70653).

df = 8946; R^2-adjusted = 0.13				
**Coefficient**	**Estimate**	**Std. error**	**t-value**	**p-value**
Intercept	-3.76	0.91	-4.11	<0.0001
Trophic level	6.59	0.21	30.70	<0.0001
SST	-0.32	0.02	-17.08	<0.0001

Table S3. Summary table for variance explained by first and second principal component (PC) of landings (obs), simulated fish populations (mod), and sea surface temperature (sst) across 55 large marine ecosystems (TL: trophic level).

		**PC1obs**	**PC2obs**	**PC1mod**	**PC2mod**	**PC1sst**	**PC2sst**
TL2	min	0.33	0.17	0.81	0.03	0.69	0.05
	max	0.52	0.34	0.93	0.10	0.89	0.11
	mean	0.43	0.26	0.88	0.06	0.78	0.09
TL3	min	0.28	0.06	0.54	0.01	0.55	0.03
	max	0.92	0.38	0.99	0.34	0.94	0.20
	mean	0.44	0.20	0.89	0.07	0.77	0.10
TL4	min	0.22	0.08	0.53	0.00	0.55	0.02
	max	0.84	0.28	0.99	0.38	0.96	0.28
	mean	0.42	0.19	0.90	0.07	0.77	0.10

Table S4. Correlations across principal components (PCs) of landings (obs), simulated fish populations (mod), and sea surface temperature (sst) across large marine ecosystems, indicated here as percentage for which Pearson's r was >0.7 ($r^2 \geq 0.5$). Numbers (1 or 2) indicate which PCs were cross-correlated; number of LMEs included for trophic level 2: n=7; trophic level 3: n=39; trophic level 4: n=55.

TL	obs1~mod1	obs2~mod2	obs1~mod2	obs2~mod1	obs1~sst1	obs2~sst2	obs1~sst2	obs2~sst1
2	29	14	14	43	71	0	0	0
3	54	13	8	26	28	8	0	3
4	62	22	11	49	15	0	5	4

Table S5. Correlations across principal components (PCs) of landings (obs), simulated fish populations (mod), and sea surface temperature (sst) across large marine ecosystems, indicated here as percentage for which Pearson's r was >0.9 ($r^2 \geq 0.8$). Numbers (1 or 2) indicate which PCs were cross-correlated; number of LMEs included for trophic level 2: n=7; trophic level 3: n=39; trophic level 4: n=55.

TL	obs1~mod1	obs2~mod2	obs1~mod2	obs2~mod1	obs1~sst1	obs2~sst2	obs1~sst2	obs2~sst1
2	0	0	0	0	0	0	0	0
3	21	0	0	3	0	0	0	0
4	29	2	0	9	0	0	0	0

Supplementary references

1 Heessen, H. J. L., Daan, N. & Ellis, J. R. *Fish atlas of the Cebtic Sea, North Sea, and Baltic Sea*. (KNNV Publishing and Wageningen Academic Publishers, 2015).

2 Froese, R. & Pauly, D. *FishBase, version (01/2021)* 2021).

3 Taylor, C. C. Cod growth and temperature. *ICES Journal of Marine Science* **23**, 366-370 (1958).

4 Rountrey, A. N., Coulson, P. G., Meeuwig, J. J. & Meekan, M. Water temperature and fish growth: otoliths predict growth patterns of a marine fish in a changing climate. *Global Change Biology* **20**, 2450-2458 (2014).

5 Thresher, R. E., Koslow, J. A., Morison, A. K. & Smith, D. C. Depth-mediated reversal of the effects of climate change on long-term growth rates of exploited marine fish. *Proceedings of the National Academy of Sciences of the United States of America* **104**, 7461-7465 (2007).

6 Neuheimer, A. B., Thresher, R. E., Lyle, J. M. & Semmens, J. M. Tolerance limit for fish growth exceeded by warming waters. *Nature Climate Change* **1**, 110-113 (2011).

7 Martino, J. C., Fowler, A. J., Doubleday, Z. A., Grammer, G. L. & Gillanders, B. M. Using otolith chronologies to understand long-term trends and extrinsic drivers of growth in fisheries. *Ecosphere* **10** (2019).

8 Smolinski, S. Sclerochronological approach for the identification of herring growth drivers in the Baltic Sea. *Ecological Indicators* **101**, 420-431 (2019).

9 Grammer, G. L. *Using biogeochemical tracers and sclerochronologies derived from fish otoliths to detect environmental change* PhD Thesis thesis, The University of Adelaide, (2015).

10 Gillanders, B. M., Black, B. A., Meekan, M. G. & Morrison, M. A. Climatic effects on the growth of a temperate reef fish from the Southern Hemisphere: a biochronological approach. *Marine Biology* **159**, 1327-1333 (2012).

11 von Biela, V. R. *et al.* Evidence of bottom-up limitations in nearshore marine systems based on otolith proxies of fish growth. *Marine Biology* **162**, 1019-1031 (2015).

12 Matta, M. E., Black, B. A. & Wilderbuer, T. K. Climate-driven synchrony in otolith growth-increment chronologies for three Bering Sea flatfish species. *Marine Ecology Progress Series* **413**, 137-145 (2010).

13 Ong, J. J. L. *et al.* Evidence for climate-driven synchrony of marine and terrestrial ecosystems in northwest Australia. *Global Change Biology* **22**, 2776-2786 (2016).

14 Coulson, P. G., Black, B. A., Potter, I. C. & Hall, N. G. Sclerochronological studies reveal that patterns of otolith growth of adults of two co-occurring species of Platycephalidae are synchronised by water temperature variations. *Marine Biology* **161**, 383-393 (2014).

15 Black, B. A., Matta, M. E., Helser, T. E. & Wilderbuer, T. K. Otolith biochronologies as multidecadal indicators of body size anomalies in yellowfin sole (*Limanda aspera*). *Fisheries Oceanography* **22**, 523-532 (2013).

16 Matta, M. E., Helser, T. E. & Black, B. A. Otolith biochronologies reveal latitudinal differences in growth of Bering Sea yellowfin sole *Limanda aspera*. *Polar Biology* **39**, 2427-2439 (2016).

17 Ong, J. J. L. *et al.* Contrasting environmental drivers of adult and juvenile growth in a marine fish: implications for the effects of climate change. *Scientific Reports* **5** (2015).

18 Ong, J. J. L. *et al.* Cross-continent comparisons reveal differing environmental drivers of growth of the coral reef fish, *Lutjanus bohar*. *Coral Reefs* **36**, 195-206 (2017).

19 Black, B. A., Allman, R. J., Schroeder, I. D. & Schirripa, M. J. Multidecadal otolith growth histories for red and gray snapper (*Lutjanus* spp.) in the northern Gulf of Mexico, USA. *Fisheries Oceanography* **20**, 347-356 (2011).

20 Dzaugis, M. P., Allman, R. J. & Black, B. A. Importance of the spring transition in the northern Gulf of Mexico as inferred from marine fish biochronologies. *Marine Ecology Progress Series* **565**, 149-162 (2017).

21 Vieira, A. R., Dores, S., Azevedo, M. & Tanner, S. E. Otolith increment width-based chronologies disclose temperature and density-dependent effects on demersal fish growth. *Ices Journal of Marine Science* **77**, 633-644 (2020).

22 Wilhelm, M. R. *et al.* Northern Benguela *Merluccius paradoxus* annual growth from otolith chronologies used for age verification and as indicators of fisheries-induced and environmental changes. *Frontiers in Marine Science* **7** (2020).

23 Smolinski, S. & Mirny, Z. Otolith biochronology as an indicator of marine fish responses to hydroclimatic conditions and ecosystem regime shifts. *Ecological Indicators* **79**, 286-294 (2017).

24 Barrow, J., Ford, J., Day, R. & Morrongiello, J. Environmental drivers of growth and predicted effects of climate change on a commercially important fish, *Platycephalus laevigatus*. *Marine Ecology Progress Series* **598**, 201-212 (2018).

25 Morrongiello, J. R. & Thresher, R. E. A statistical framework to explore ontogenetic growth variation among individuals and populations: a marine fish example. *Ecological Monographs* **85**, 93-115 (2015).
26 van der Sleen, P. *et al.* Otolith increments in European plaice (*Pleuronectes platessa*) reveal temperature and density-dependent effects on growth. *ICES Journal of Marine Science* **75**, 1655-1663 (2018).
27 Nguyen, H. M. *et al.* Growth of a deep-water, predatory fish is influenced by the productivity of a boundary current system. *Scientific Reports* **5** (2015).
28 van der Sleen, P. *et al.* Long-term Bering Sea environmental variability revealed by a centennial-length biochronology of Pacific ocean perch *Sebastes alutus*. *Climate Research* **71**, 33-45 (2017).
29 Thompson, J. E. & Hannah, R. W. Using cross-dating techniques to validate ages of aurora rockfish (*Sebastes aurora*): estimates of age, growth and female maturity. *Environmental Biology of Fishes* **88**, 377-388 (2010).
30 Black, B. A. *et al.* Winter and summer upwelling modes and their biological importance in the California Current Ecosystem. *Global Change Biology* **17**, 2536-2545 (2011).
31 Black, B. A., Boehlert, G. W. & Yoklavich, M. M. Establishing climate-growth relationships for yelloweye rockfish (*Sebastes ruberrimus*) in the northeast Pacific using a dendrochronological approach. *Fisheries Oceanography* **17**, 368-379 (2008).
32 Mazloumi, N., Burch, P., Fowler, A. J., Doubleday, Z. A. & Gillanders, B. M. Determining climate-growth relationships in a temperate fish: A sclerochronological approach. *Fisheries Research* **186**, 319-327 (2017).
33 Tanner, S. E. *et al.* Regional climate, primary productivity and fish biomass drive growth variation and population resilience in a small pelagic fish. *Ecological Indicators* **103**, 530-541 (2019).
