# Supplementary material for: Interannual temperature variability is a principal driver of low-frequency fluctuations in marine fish populations
Source: Commun Biol. 2022 Jan 11;5:28. doi: 10.1038/s42003-021-02960-y (PMC8752724; doi:10.1038/s42003-021-02960-y)
Supplement: Supplementary file 2 — Description of Additional Supplementary Files [file 42003_2021_2960_MOESM2_ESM.pdf]

Description of Additional Supplementary Data Files

File name: Supplementary Data 1

Description: Biological time series shown in in figure 1.

File name: Supplementary Data 2

Description: Sea surface temperature data shown in figure 1.

File name: Supplementary Data 3

Description: Stock size estimates of fish from the North Sea and Celtic Sea used in figure S1.

File name: Supplementary Data 4

Description: Fish life-history data used in figure S2 and table S2.

File name: Supplementary Data 5

Description: R-scripts used for the model simulations shown in figures 1, 2 and 5.
