# Supplementary material for: Interannual temperature variability is a principal driver of low-frequency fluctuations in marine fish populations
Source: Commun Biol. 2022 Jan 11;5:28. doi: 10.1038/s42003-021-02960-y (PMC8752724; doi:10.1038/s42003-021-02960-y)
Supplement: Supplementary file 8 — Reporting Summary [file 42003_2021_2960_MOESM8_ESM.pdf]

nature research

Corresponding author(s): Peter van der Sleen
Last updated by author(s): Nov 8, 2021

Reporting Summary

Nature Research wishes to improve the reproducibility of the work that we publish. This form provides structure for consistency and transparency in reporting. For further information on Nature Research policies, see our Editorial Policies and the Editorial Policy Checklist.

Statistics

For all statistical analyses, confirm that the following items are present in the figure legend, table legend, main text, or Methods section.

n/a	Confirmed	
☐	☒	The exact sample size (*n*) for each experimental group/condition, given as a discrete number and unit of measurement
☐	☒	A statement on whether measurements were taken from distinct samples or whether the same sample was measured repeatedly
☐	☒	The statistical test(s) used AND whether they are one- or two-sided<br>*Only common tests should be described solely by name; describe more complex techniques in the Methods section.*
☐	☒	A description of all covariates tested
☐	☒	A description of any assumptions or corrections, such as tests of normality and adjustment for multiple comparisons
☐	☒	A full description of the statistical parameters including central tendency (e.g. means) or other basic estimates (e.g. regression coefficient) AND variation (e.g. standard deviation) or associated estimates of uncertainty (e.g. confidence intervals)
☐	☒	For null hypothesis testing, the test statistic (e.g. *F*, *t*, *r*) with confidence intervals, effect sizes, degrees of freedom and *P* value noted<br>*Give P values as exact values whenever suitable.*
☒	☐	For Bayesian analysis, information on the choice of priors and Markov chain Monte Carlo settings
☐	☒	For hierarchical and complex designs, identification of the appropriate level for tests and full reporting of outcomes
☐	☒	Estimates of effect sizes (e.g. Cohen's *d*, Pearson's *r*), indicating how they were calculated

Our web collection on statistics for biologists contains articles on many of the points above.

Software and code

Policy information about availability of computer code

Data collection	This study uses models that are parametrized based on open-access or published fish life-history data and open-access climate data. The models are validated using open-access or published fisheries catch data. A data availability statement is included.
Data analysis	All analyses where preformed in R. Detailed model description is provided in the manuscript and relevant R-code included.

For manuscripts utilizing custom algorithms or software that are central to the research but not yet described in published literature, software must be made available to editors and reviewers. We strongly encourage code deposition in a community repository (e.g. GitHub). See the Nature Research guidelines for submitting code & software for further information.

Data

Policy information about availability of data

All manuscripts must include a data availability statement. This statement should provide the following information, where applicable:

- Accession codes, unique identifiers, or web links for publicly available datasets
- A list of figures that have associated raw data
- A description of any restrictions on data availability

All data needed to evaluate the conclusions in the paper are either publicly available or can be found in the work cited. The data shown in figure 1, S1 and S2 are also included as supplementary data files. The R-scripts used for the model simulations (e.g. Fig 1, 2 and 5) are included as supplementary files. All other R-scripts used are available upon request.

Field-specific reporting

Please select the one below that is the best fit for your research. If you are not sure, read the appropriate sections before making your selection.

☐ Life sciences ☐ Behavioural & social sciences ☒ Ecological, evolutionary & environmental sciences

For a reference copy of the document with all sections, see nature.com/documents/nr-reporting-summary-flat.pdf

Ecological, evolutionary & environmental sciences study design

All studies must disclose on these points even when the disclosure is negative.

Study description	Modeling low-frequency dynamics of marine fish populations by considering environmental- and demographic factors.
Research sample	Global 1x1 degree sea surface temperature (~1950-2018) and Net Primary Productivity data (2003-2018). Global database of fisheries landings (3789 species time series from 1955-2014). Life history data (maximum age and tropic position) for 3917 fish species. Abundance data for 60 species from the North Sea and Celtic Sea (~1977-2013).
Sampling strategy	Not applicable
Data collection	Not applicable
Timing and spatial scale	Global; from 1955 to present.
Data exclusions	Fish species excluded in the analysis presented in figure S1 are described in figure caption. In none of the other analyses data was excluded.
Reproducibility	Data and R-scripts are provided to reproduce the results presented.
Randomization	Not applicable
Blinding	Not applicable

Did the study involve field work? ☐ Yes ☒ No

Reporting for specific materials, systems and methods

We require information from authors about some types of materials, experimental systems and methods used in many studies. Here, indicate whether each material, system or method listed is relevant to your study. If you are not sure if a list item applies to your research, read the appropriate section before selecting a response.

Materials & experimental systems

n/a	Involved in the study	
☒	☐	Antibodies
☒	☐	Eukaryotic cell lines
☒	☐	Palaeontology and archaeology
☒	☐	Animals and other organisms
☒	☐	Human research participants
☒	☐	Clinical data
☒	☐	Dual use research of concern

Methods

n/a	Involved in the study	
☒	☐	ChIP-seq
☒	☐	Flow cytometry
☒	☐	MRI-based neuroimaging
